# ^18^F-FDG PET/CT revealed sporadic schwannomatosis involving the lumbar spinal canal and both lower limbs: a case report

**DOI:** 10.3389/fmed.2024.1346647

**Published:** 2024-03-20

**Authors:** Xiaotian Li, Xianwen Hu, Pan Wang, Jiong Cai

**Affiliations:** ^1^Department of Nuclear Medicine, Affiliated Hospital of Zunyi Medical University, Zunyi, China; ^2^Department of Nuclear Medicine, People’s Hospital of Qianxinan Buyi and Miao Minority Autonomous Prefecture, Xingyi, China

**Keywords:** schwannomatosis, ^18^F-FDG, PET/CT, neurofibromatosis, schwannoma

## Abstract

Schwannomatosis is a rare autosomal dominant hereditary syndrome disease characterized by multiple schwannomas throughout the body, without bilateral vestibular schwannoma or dermal schwannoma. The most common location of schwannomatosis is the head and neck, as well as the limbs, while multiple schwannomas in the lumbosacral canal and lower extremities are relatively rare. In this study, we report a 79-year-old woman diagnosed with schwannomatosis. MRI and contrast-enhanced imaging revealed multiple schwannomas in both lower extremities. An ^18^F-FDG PET/CT examination revealed that in addition to multiple tumors with increased ^18^F-FDG uptake in both lower extremities, there was also an increased ^18^F-FDG uptake in a mass in the lumbosacral canal. These masses were confirmed to be schwannomas by pathology after surgery or biopsy. ^18^F-FDG PET/CT findings of schwannomas were correlated with MRI and pathological components. Antoni A area rich in tumor cells showed significant enhancement on contrast-enhanced T1WI, and PET/CT showed increased uptake of ^18^F-FDG in the corresponding area, while Antoni B region rich in mucus showed low enhancement on contrast-enhanced T1WI, accompanied by a mildly increased ^18^F-FDG uptake.

## Introduction

Schwannomatosis is a very rare disease with an incidence of 0.58 cases per 1 million people per year (1, 2), which was officially named in 1973 by Niimura (3). In 1997, the National Institutes of Health classified schwannomatosis as the third subtype of neurofibromatosis, namely NF3 (4, 5). It differs from types 1 and 2 neurofibromatosis in genetics, pathology, and clinical features, but due to insufficient understanding of the disease, it is often misdiagnosed clinically as one of the two subtypes (6). NF1 is mainly characterized by multiple café-au-lait spots on the skin, retinal iris patches, subcutaneous, or fascicular neurofibromas, and its occurrence is related to the loss of the NF1 tumor suppressor gene mutation (7, 8). NF2 is mainly characterized by bilateral vestibular schwannomas and can be accompanied by cataracts and tumors in other parts such as the brain, spinal cord, and periphery, and its occurrence is mainly caused by germline or somatic mutations and deletions of the NF2 gene (9, 10). Schwannomatosis (NF3) is characterized by having multiple schwannomas throughout the body without bilateral vestibular schwannomas or dermal schwannomas, and according to whether the tumors have a genetic basis, which can be divided into sporadic and familial forms (11). Among them, familial cases account for 13–25% of schwannomatosis, most of which are characterized by multiple peripheral nerve or spinal nerve root schwannomas (2, 12). Sporadic cases account for the majority of schwannomatosis, and there is no familial clustering or genetic susceptibility (13). In this study, we report a rare sporadic case of schwannomatosis with pathologic and genetic diagnostic support. The aim is to share our experience in the clinical diagnosis and treatment of schwannomatosis to better understand the rare entity of sporadic schwannomatosis.

## Case description

**Figure 1:**
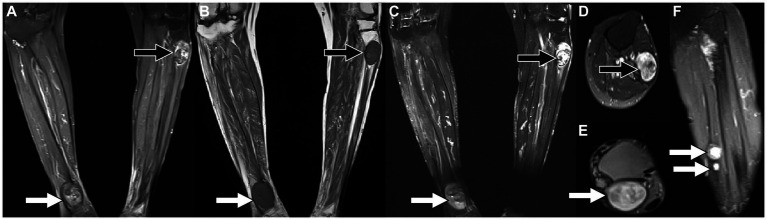
Magnetic resonance imaging [MRI; **(A)** T_2_-weighted imaging (T_2_WI); **(B)** T_1_-weighted imaging (T_2_WI)] reveals that there are fusiform soft tissue masses with low signal on T_1_WI and high signal on T_2_WI under the skin of the proximal tibia on the left and the distal tibia on the right, of which boundaries are clear. Contrast-enhanced T1WI sequence scan after contrast agent injection [**(C)** Coronal; **(D,E)** Axial; **(F)** Sagittal view shows small nodules of markedly enhanced soft tissue in the middle of the left calf (arrows)] revealed significant enhancement in both lower extremity lesions.

**Figure 2:**
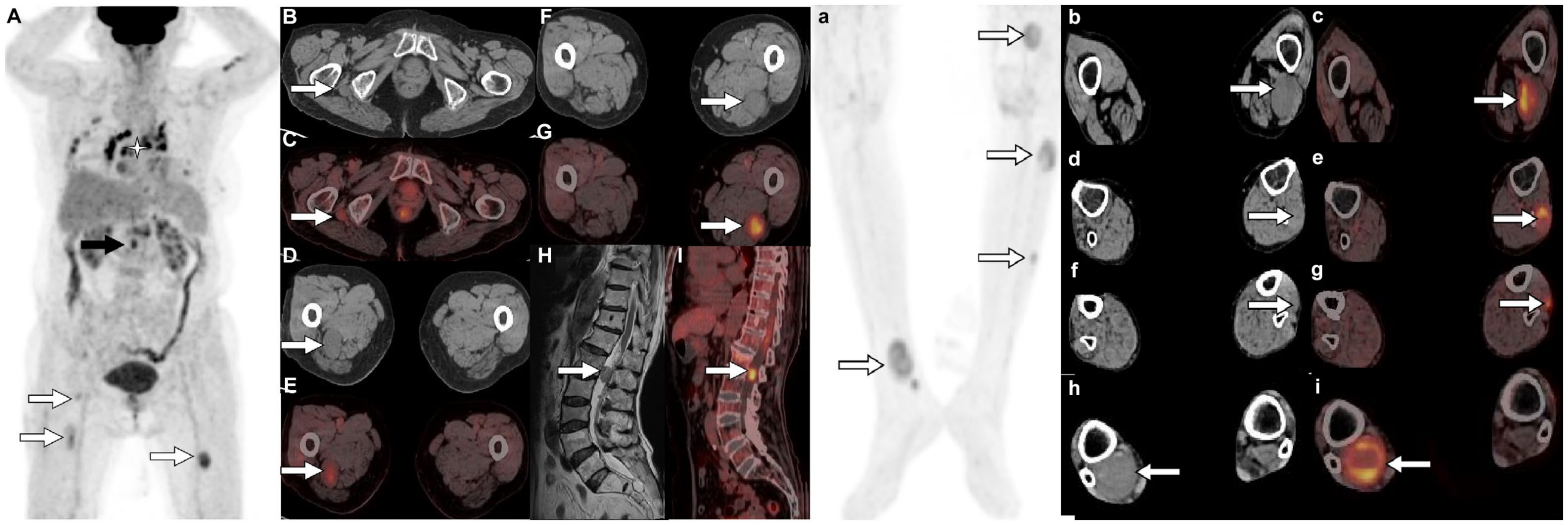
^18^F-FDG PET/CT [**(A,a)** Maximum intensity projection (MIP)] was subsequently performed to further evaluate the patient’s whole-body condition and revealed multiple nodules of hypermetabolic uptake in the lumbar spinal canal (black arrow) and lower limbs (white arrows). Besides, multiple high metabolic nonspecific lymph node uptakes were observed in the mediastinum (asterisk arrow). Tomographic images show multiple soft tissue density nodules with high FDG uptake on the lateral side of the right ischial tuberosity [**(B)** CT; **(C)** PET/CT fusion; arrows], upper thigh [**(D)** CT; **(E)** PET/CT fusion; arrows], middle [**(F)** CT; **(G)** PET/CT fusion; arrows] and lower left thigh [**(b)** CT; **(c)** PET/CT fusion; arrows], upper [**(d)** CT; **(e)** PET/CT fusion; arrows] and middle leg [**(f)** CT; **(g)** PET/CT fusion; arrows; SUVmax 7.5], and lower right leg [**(h)** CT; **(i)** PET/CT fusion; arrows]. In addition, isomuscular signal on T1WI (**H**, arrow) and high metabolic uptake nodule on PET/CT (**I**, arrow) were also observed in the posterior spinal canal of the first lumbar spine.

**Figure 3:**
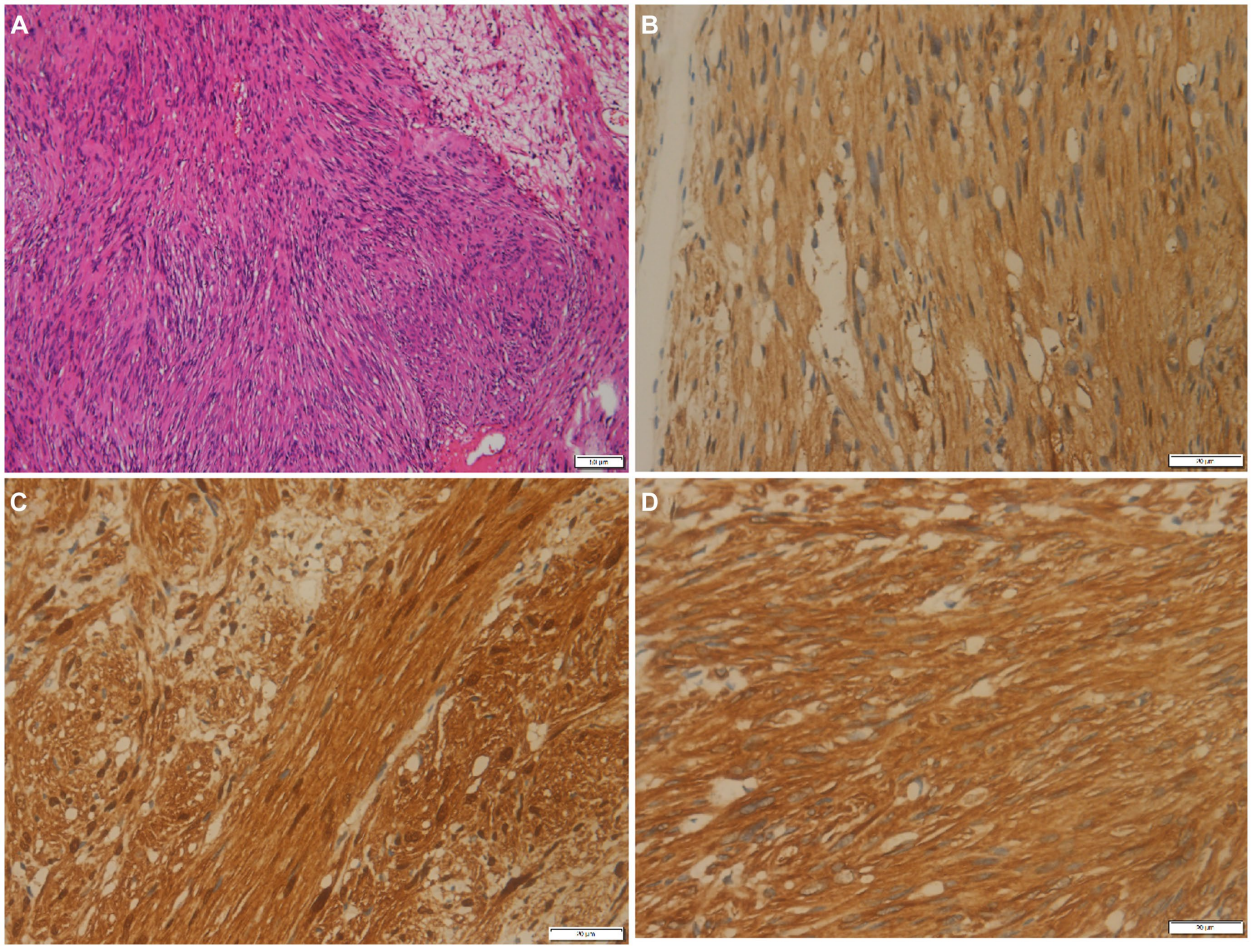
**(A)** Histological examination shows tumor cells are spindle-shaped, bundle-shaped, and focal fence-shaped (hematoxylin–eosin stain, original magnification ×100). Immunohistochemistry shows that tumor cells positively express PGP9.5 **(B)**, S100 **(C)**, and vimentin **(D)**.

A 79-year-old woman presented with painless masses on both lower limbs in May 2012. In the past 3 months, the patient found that some of these lumps were gradually increasing, and she came to the Affiliated Hospital of Zunyi Medical University for medical help in May 2021. Both the patient and her family had no history of cancer or genetic diseases. Physical examination found multiple soft tissue masses approximately 1 cm above the skin surface in both lower limbs, no ulceration or purulent secretions, and no positive signs in other parts of the body. Laboratory tests and serum tumor markers were all within the normal reference range upon admission. Low-limb magnetic resonance imaging (MRI) showed fusiform low-signal soft tissue swelling at the proximal and distal ends of the right tibia subcutaneously, with clear boundaries and significant enhancement after the injection of contrast agent, showing increased signals in lower limb lesions (Figure 1). Subsequently, the patient underwent fluorine-18-fluorodeoxyglucose positron emission tomography/computed tomography (^18^F-FDG PET/CT) to evaluate the systemic condition. The results showed that multiple ^18^F-FDG uptake nodules increased in the first lumbar vertebra, the lateral side of the right ischial tuberosity, the upper part of the thigh, the lower part of the left thigh, and the right calf (Figure 2). Moreover, additional multiple high-metabolic non-specific lymph nodes were observed in the mediastinum. Subsequently, the patient underwent bilateral resection of the lower extremity tumor and an intraspinal tumor biopsy at the level of the first lumbar spine under anesthesia. The histopathological results showed that these tumors had the same pathological properties and were all schwannomas (Figure 3). Genetic testing indicated that the patient had an SMARCB1 gene mutation in the blood. The patient received anti-inflammatory treatment for 7 days postoperatively and was discharged with a good general condition. Up to now, the patient has been followed up for 30 months, and there are no signs of tumor recurrence in the surgical area of both lower limb tumors, nor have any nerve compression symptoms such as numbness or pain in either lower limb been found.

## Discussion

Schwannomatosis is rare clinically and can occur in any age group, with the most common occurrence being between 30 and 60 years old, without significant differences in incidence rates between male and female patients (14, 15). Its characteristic feature is multiple schwannomas throughout the body without bilateral vestibular schwannomas or cutaneous neurofibromas, which belong to autosomal incomplete penetrance inheritance disease (16). The mechanism of this disease has not been thoroughly studied yet and may be related to abnormal expression of gene mutations. According to literature records, the abnormal expression of genes such as SMARCB1 (INI1), the LZTR1 gene, and the NF2 gene has been reported more frequently as possible causes of neurofibromatosis patients’ onset, among which the SMARCB1 gene is considered the susceptibility gene for neurofibromatosis (17, 18). The clinical manifestations of this disease are atypical. Symptoms of nerve compression vary depending on the location of the tumor, with neuropathic pain resulting from compression of adjacent tissues being the primary symptom. The patient we reported was a 79-year-old woman, whose primary clinical symptoms were multiple painless masses in her lower limbs that had been present for years. There are slight differences in the age of onset and clinical symptoms compared to those reported for schwannomatosis in the literature.

Schwannoma is a tumor composed of mixed Schwann cells, perineurial-like cells, and fibroblasts, with scattered nerve fibers, linear collagen bands, and mucoid matrix (19). It is mostly round or lobulated, well-defined, encapsulated, of moderate hardness or elasticity, without fluctuation, and when the larger tumor appears to have mucoid degeneration, a puncture can draw out non-coagulating bloody fluid (20). Microscopically, schwannomas show spindle-shaped tumor cells arranged in two phases: some cells are closely adjacent to nuclear palisade-like arrangements (with Verocay bodies in the Antoni A area), while other areas only see loose aggregation of cells on a mucoid background (Antoni B area) (21, 22). The lesion often exhibits prominent thick-walled blood vessels, along with frequent occurrences of mucoid degeneration, hemorrhage, and calcification within the tumor. Immunohistochemically, there was diffuse strong positive expression of S100 and SOX10 (23–25), focal positive expression of glial fibrillary acidic protein (GFAP) in some cases, focal keratin positivity in a few cases, while negative for melanocyte-specific markers HMB45 and melan-A (24, 26). The patient underwent bilateral lower limb tumor resection and intraspinal tumor biopsy at the level of the first lumbar spine, which microscopically showed that these tumors were composed of Antoni A areas rich in spindle cells and Antoni B areas rich in mucoid substance. Immunohistochemical results showed that PGP9.5, S100, and vimentin were all positive, consistent with the histopathological features of schwannoma.

The presentation of schwannomatosis frequently manifests as imaging features characteristic of schwannoma. The CT scan typically reveals a round or oval density with a well-defined boundary and a low-density shadow in some areas with cystic degeneration (27). The MRI findings of this condition are characterized by equal or low signals on T1WI and slightly high or high signals on T2WI (28, 29). Multifocal schwannoma in ^18^F-FDG PET/CT is rarely described in the literature and occurs more frequently in the head and neck, spinal canal, and upper limb (30–33). The cases we reported were located in the pelvis, lower limb, and lumbar spinal canal, which are rarely reported in the literature. These tumors have a certain correlation between ^18^F-FDG PET/CT and contrast-enhanced T1WI. The Antoni A region rich in tumor cells showed significant enhancement on contrast-enhanced T1WI, and the corresponding region presented increased ^18^F-FDG uptake on PET/CT. While Antoni B areas rich in mucoid substances show low enhancement on contrast-enhanced T1WI, accompanied by mildly increased ^18^F-FDG uptake. The comprehension of these findings is crucial for the exploration and elucidation of multifocal enhancements in ^18^F-FDG activity within schwannomas. The ^18^F-FDG uptake of schwannomas varies greatly with the components of the tumor. Moreover, our case showed that ^18^F-FDG PET/CT can also accurately locate each tumor in schwannomatosis, and more hidden lesions can be found. In addition, the diagnosis of schwannomatosis should exclude the presence of vestibular schwannoma, requiring an MRI examination for confirmation.

According to the diagnostic criteria for schwannomatosis developed by Mac Collin et al. (34), which include: (1) A confirmed diagnosis is defined as (a) age over 30 years and presence of at least two non-cutaneous neurofibromas, with at least one confirmed by pathological examination; absence of vestibular schwannoma confirmed by high-resolution MRI, and no constitutive mutation in the NF2 gene. (b) A pathologically proven schwannoma or meningioma and a first-degree relative with schwannomatosis. (2) Possible diagnoses: (a) age below 30 years or above 45 years and two or more non-intradermal schwannomas confirmed by pathological examination as schwannoma, without abnormal symptoms of the eighth cranial nerve function, and without constitutive mutations in the NF2 gene. (b) Imaging confirms non-vestibular schwannoma and one first-degree relative meeting the diagnostic criteria. The diagnosis of segmental schwannomatosis requires meeting definite or possible schwannomatosis diagnostic criteria limited to one limb or five adjacent spinal segments. Based on these diagnostic criteria, the patient we reported is consistent with the diagnosis of schwannomatosis.

The clinical manifestations and signs of this disease are very similar to NF1 and NF2, and consequently, they need to be distinguished from them. NF1 is characterized by coffee-au-Lait plaques, subcutaneous nodules, and Lisch nodules, while NF2 is characterized by bilateral vestibular schwannomas, which can be differentiated from schwannomatosis (35). High-resolution MRI (internal auditory canal thickness below 3 mm) confirmed the absence of bilateral vestibular schwannomas in the patient, so we highly suspected the diagnosis of schwannomatosis. Schwannomatosis is generally easier to distinguish from NF1 and simple NF2, but it is difficult to differentiate from mosaic NF2. Therefore, additional genetic testing was conducted on the excised tumors, revealing a mutation in the SMARCB1 (blood) gene, which validated our diagnosis.

The SMARCB1 gene (also known as INI1) is a tumor suppressor gene, whose mutation is associated with neurofibromatosis type 2 and schwannomatosis (36). The SMARCB1 gene is located at 22q11.23 and contains nine exons that encode the SMARCB1 protein, which has a tumor-suppressive function (37). Currently, more commonly reported genes that may lead to the development of schwannomatosis include the SMARCB1 (INI1) gene (38), the LZTR1 gene (39), and the NF2 gene (40). Among them, the SMARCB1 gene was first reported by scholars to be a susceptibility gene for schwannomatosis (5), and it was found that mutations in the SMARCB1 gene play a more prominent role in the pathogenesis of schwannomatosis (2). Moreover, many scholars believe that germline mutations in the SMARCB1 gene leading to abnormalities in the structure and functional expression of the protein may be the main cause of the development of schwannomatosis (41). Current studies show that mutations in this gene occur in 8–10% of sporadic schwannomatosis patients and 40–50% of familial schwannomatosis patients (11, 42).

The main treatment for schwannomatosis is surgical resection (43, 44). However, not all schwannomas necessitate surgery; it is typically reserved for patients with symptoms such as bleeding, pain, neurological dysfunction, and a tendency to malignant transformation (45–47). The main problem facing neurosurgeons today is to achieve complete tumor resection and prevent recurrence while maximizing the protection of neurological function under the premise of protecting nerve function (47). The surgical treatment of schwannomas depends on the size, location, and complexity of the tumor and should comprehensively consider the individual situation of the patient and implement the most appropriate personalized surgical plan for the patient. The majority of schwannomatosis cases are benign, thus indicating a favorable prognosis. Due to the small volume of the tumor in our patient’s lumbar spinal canal and the absence of corresponding clinical manifestations, further resection of the tumor in the lumbar spinal canal was not performed following bilateral lower limb tumor resection and lumbar spinal canal tumor puncture biopsy. The patient has a good prognosis, and no disease progression was observed during the follow-up period.

## Conclusion

^18^F-FDG PET/CT contributes to the accurate localization of schwannomatosis and has certain imaging characteristics; namely, the Antoni A area rich in tumor cells showed increased uptake of ^18^F-FDG, while the Antoni B region rich in mucus only showed a mildly increased ^18^F-FDG uptake.

## Data availability statement

The original contributions presented in the study are included in the article/supplementary material, further inquiries can be directed to the corresponding author.

## Ethics statement

Written informed consent was obtained from the individual(s) for the publication of any potentially identifiable images or data included in this article.

## Author contributions

XL: Writing – original draft, Resources, Methodology, Investigation, Project administration. XH: Formal analysis, Data curation, Conceptualization, Writing – original draft, Resources, Methodology. PW: Writing – review & editing, Visualization, Validation, Supervision, Software. JC: Project administration, Data curation, Conceptualization, Writing – review & editing, Visualization.
